# Dental age estimation in children affected by juvenile rheumatoid arthritis

**DOI:** 10.1007/s00414-020-02395-w

**Published:** 2020-08-20

**Authors:** Vilma Pinchi, Ilenia Bianchi, Francesco Pradella, Giulia Vitale, Martina Focardi, Ingrid Tonni, Luigi Ferrante, Andrea Bucci

**Affiliations:** 1 Department of Health Sciences, Section of Forensic Medical Sciences, University of Florence, largo Brambilla 3, 50134 Florence, Italy; 2 Dental School, Department of Medical and Surgical Specialties, Radiological Sciences and Public Health, University of Brescia, P.le Spedali Civili, 25123 Brescia, Italy; 3 Center of Epidemiology, Biostatistics and Medical Information Technology, Department of Biomedical Sciences and Public Health, Università Politecnica delle Marche, Via Tronto 10/a, 60020 Ancona, Italy; 4 Department of Economics, Università degli Studi G. d’Annunzio Chieti-Pescara, Viale Pindaro 42, 65127 Pescara, Italy

**Keywords:** Juvenile rheumatoid arthritis, Dental age estimation, Dental maturation, Forensic odontology

## Abstract

**Abstract:**

Dental root calcification has proven to be a reliable biological evidence to estimate chronological age of children. The development of structures usually examined in the age estimation forensic practice (e.g. skeleton, teeth) is supposed to be influenced by diseases and nutritional, environmental, ethnic, and ultimately even socioeconomic factors. This research aims to study the age estimation in children affected by juvenile rheumatoid arthritis (JRA) with and without steroids treatment and compared with healthy subjects.

**Material and methods:**

Dental age estimations based on 752 OPGs, 420 girls and 332 boys, aged from 3.3 to 15.99 years, were provided by applying Demirjian and Willems’ original methods. Of the whole sample, 103 individuals were affected by JRA and 40 received a continuous corticosteroid therapy, over 1 year long.

**Conclusions:**

Willems’ and Demirjian’s original methods, as methods commonly applied to estimate age for sub-adults with unremarkable medical history, can be used for medico-legal purposes to children affected by JRA. Willems’ method tended to underestimate age while Demirjian’s method resulted to be prone to overestimation for both healthy and JRA-affected children. JRA showed to have no influence on root calcification process even in children that received steroid treatment for 1 year or longer.

**Electronic supplementary material:**

The online version of this article (10.1007/s00414-020-02395-w) contains supplementary material, which is available to authorized users.

## Introduction

The forensic age estimation procedures usually involve the analysis of the skeletal and dental maturation. Dental age estimation is a reliable and widely performed forensic practice and the methods adopted in children mainly rely on the permanent teeth developmental changes based on dental crown and root mineralization. The developmental process of the other structures usually examined in the age estimation forensic practice, i.e. the skeleton, is deemed influenced by diseases and nutritional, environmental, ethnic and ultimately even socioeconomic factors.

An impairment in bone growth (delay) is a well-recognized complication in children suffering from juvenile rheumatoid arthritis (JRA) [1, 2]. Several factors have been mentioned in the literature as the possible cause of growth retardation in JRA, including nutritional abnormalities, decreased growth factor synthesis, the inflammatory process, action of cytokines, drug effects and, among them, especially the use of corticosteroids in case of prolonged therapies [3–8].

Very few researches, however, focused on the possible influence of such a disease on the dental maturation process of affected children compared with the non-affected individuals. The research wishes to provide a contribution to the comprehension of the independence of the dental maturation process from the skeletal development even in individuals affected by diseases otherwise usually jeopardizing the overall bone and body growth and even the cranio-facial growth.

To this purpose, the research aims to study the age estimation in JRA-affected children, with and without steroids treatment. Secondly the research is addressed to disclose difference in age estimation between the JRA-affected children compared with the healthy subjects.

## Materials and methods

The examined sample is composed of a total of 752 OPGs (orthopantomographies), 420 girls (mean chronological age 11.83 years, range 3.3–15.98 years) and 332 boys (mean of chronological age 12.42 years, range 4.63–15.99 years). Of the whole sample, 103 individuals are affected by JRA. A sub-sample of 40 individuals received a continuous corticosteroid therapy, more than 1 year long. Another sub-sample of 63 JRA-affected individuals received no steroid therapy. The OPGs were taken for clinical purposes with the approval of the patients’ parents and the analysis of the affected people has been made with the previous approval of the local Ethics Committee.

The inclusion criteria were:

- OPGs of good quality
- Unremarkable medical history, except for the JRA in affected children
- Known date of birth and of the OPG examination

The following samples were excluded:

- OPGs belonging to children younger than 3 years or older than 16 years, according to the lower and upper limit age provided by Demirjian and Willems’ methods applied here to estimate age.

The comparison between the estimation errors between the JRA-affected and non-affected children were made, balancing the entire sample to be evenly distributed in terms of age and presence of JRA. The balanced sample is composed of a total of 493 OPGs, 265 girls and 228 boys.

The collected OPGs have been submitted to an expert forensic odontologist who was informed only about the sex of the child and worked without any knowledge of the age and other clinical information of the patients.

One classification of the dental maturation (Demirjian) and two different conversion tables have been adopted to estimate the dental age: the original Demirjian’s method based on seven teeth and Willems’ method. An intra- and inter-observer reliability assessment have been made on a sample of 40 OPGs randomly selected from healthy individuals and 35 OPGs randomly selected from JRA-affected individuals by the same forensic odontologist and by a second one after an interval of 2 weeks.

## Statistical analysis

The reliability of Demirjian and Willems’ methods was determined using the Intraclass Correlation Coefficient (ICC) for one-way model; see Shrout and Fleiss (1979) [9] and McGraw and Wong (1996) [10]. The analysis concerned two types of error evaluated for each method. Firstly, the age estimation error, *E*, was considered as the difference between the estimated and the chronological age. This measure allowed us to assess the appropriateness of the model, since its distribution indicates whether the errors are randomly dispersed. Then, absolute value of *E* (|퐸|) was analysed, in order to understand whether the size of the error changed according to the characteristics of the sample and the method applied.

A descriptive analysis of the errors, *E*_*i*_, *i = 1*,*…n*, was performed to compare subjects with different characteristics. We firstly reported the mean values and the standard deviation of *E* in JRA-affected, stratifying by sex and presence of the treatment. The statistically significant differences among the errors on the basis of the considered variables were investigated through *t* statistics. Since the distributions of the absolute value of *E* were asymmetric, the statistical analysis was performed through a non-parametric approach, and the median value and the first and third quartile were reported. In order to achieve the second goal of this study, we compared the mean and standard deviation both of *E* and absolute value of *E* stratifying the balanced sample by gender and presence of JRA.

A linear regression model was used to estimate the effect of the treatment on the age estimation error, *E*, in the sample of subjects affected by JRA.

The association of *E* with the gender, the presence of JRA and the age of the subject in the balanced sample were estimated through a linear regression model for both Demirjian and Willems’ methods.

Quantile regression was used to evaluate the association of the absolute value of *E* with the following characteristics of the subject: sex, presence of treatment and age. We repeated the quantile regression analysis on the balanced sample with the following covariates: sex, presence of JRA and age. Three quartiles of the distributions of the absolute errors were evaluated. The results were shown in a plot where the x-axis indicated the three quartiles of the absolute error and the y-axis exhibited the effect of each variable (regression coefficients) and 95% confidence bands (grey area). If the confidence interval included the zero, the estimates could not be considered statistically different from zero.

*p* values less than 0.05 were interpreted as indicative of statistically significant differences.

Statistical analyses were performed with the R statistical program [11].

## Results

The ICC one-way with its confidence interval at 95% level is shown in Table 1.

**Table 1:** Reliability of Demirjian and Willems’ methods using Intraclass Correlation Coefficient (ICC) for one-way model stratified by presence of disease

	Within		Between	
	Non-affected	JRA affected	Non-affected	JRA affected
Demirjian	0.92 (0.86; 0.96)	0.73 (0.54; 0.86)	0.91 (0.85; 0.95)	0.71 (0.50;0.84)
Willems	0.88 (0.78; 0.93)	0.78 (0.61; 0.88)	0.85 (0.73; 0.92)	0.77 (0.60; 0.88)

The confidence interval at 95% is reported between brackets

The intra-rater and inter-rater reliabilities were both good, regardless the affection by JRA and the method applied, with values ranging from 0.71 to 0.92.

Table 2 shows the comparison of error in chronological age estimation, as simple differences (Panel A) and absolute values (Panel B), in JRA-affected subjects in terms of gender and treatment received. Willems’ method underestimated the chronological age both in girls and boys in Panel A, which was otherwise overestimated by Demirjian’s method. The *p* values in the column showed that there were no differences between girls and boys in terms of age estimation accuracy either with Demirjian or Willems’ method, while the error was significantly different between the methods. There were no differences in the estimation of treated or untreated subjects with Demirjian and Willems’ methods. Applying Demirjian’s method, estimations resulted statistically different from Willems’ either in treated or untreated subjects.

**Table 2:** Summary statistics of the difference and the absolute value of the difference between the estimated age and the chronological age in JRA-affected subjects

Panel A: age estimation error, *E*					
Variable		*n* (%)	Demirjian mean (s.d.)	Willems mean (s.d.)	*p* Value
Sex	Female	79 (77)	0.54 (0.94)	− 0.32 (1.03)	< 0.01
	Male	24 (23)	0.27 (0.74)	− 0.17 (0.97)	0.03
		*p* Value	0.14	0.51	
Under treatment	Yes	40 (39)	0.59 (0.93)	− 0.35 (1.01)	< 0.01
	No	63 (61)	0.41 (0.88)	− 0.25 (1.02)	< 0.01
		*p* Value	0.35	0.77	
Panel B: absolute age estimation error, |*E*|					
Variable			Demirjian median (1st–3rd quartile)	Willems median (1st–3rd quartile)	*p* Value
Sex	Female		0.81 (0.27–1.00)	0.84 (0.36–1.14)	0.72
	Male		0.54 (0.14–0.71)	0.69 (0.28–0.70)	0.31
		*p* Value	0.03	0.17	
Under treatment	Yes		0.86 (0.27–1.13)	0.86 (0.55–1.10)	0.72
	No		0.67 (0.19–0.81)	0.76 (0.24–1.05)	0.30
		*p* Value	0.06	0.16	

The *p* value refers to the significance of a *t* statistics in panel A and to the significance of Wilcoxon statistics in Panel B

Panel B of Table 2 further reports the comparison of the absolute errors of estimates of chronological age, performed by non-parametric tests. No statistically significant difference was found between the methods both for boys and girls applying Demirjian’s method, while a statistically significant difference was detected among boys and girls.

No difference was found between treated and untreated subjects.

**Fig. 1:**
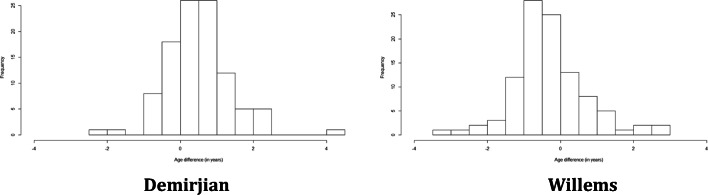
Distribution of the error in age estimation stratified by method in the JRA-affected sample

Figure 1 reports the distribution of the error in estimation stratified by the method used for age estimation in JRA-affected subjects. The errors seemed to be normally distributed, as it is also confirmed by the non-rejection of the null hypothesis of normality in the Kolmogorov-Smirnov test, which provided a *p* value of 0.30 for the errors with Demirjian and 0.29 with Willems. Hence, a linear regression model could be implemented to analyse the association of the error with the characteristics of the subjects, and the results are shown in Table 3.

**Table 3:** Estimation results of the linear regression for the difference between Demirjian and Willems’ estimated age and chronological age in subjects with JRA

	Demirjian		Willems	
Variable	Coef. (std. error)	*p* Value	Coef. (std. error)	*p* Value
Sex (male)	−0.263 (0.211)	0.216	0.147 (0.239)	0.540
Under treatment (yes)	0.157 (0.186)	0.400	− 0.093 (0.210)	0.658
Age	0.002 (0.027)	0.929	− 0.001 (0.030)	0.975

The dependent variable in the linear regression was the estimation error while the covariates were sex (with male as reference), presence of treatment (with treatment received as reference) and age. Neither sex, steroid therapy nor the chronological age resulted to be significantly associated with error.

**Fig. 2:**
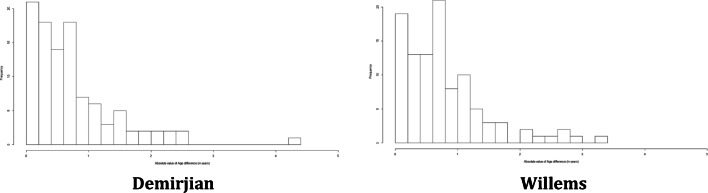
Distribution of the absolute value of the error in age estimation stratified by method of age estimation

Since the distribution of the absolute error was highly asymmetric and not normal, as shown in Fig. 2, we assessed its association with the covariates through a quantile regression.

**Fig. 3:**
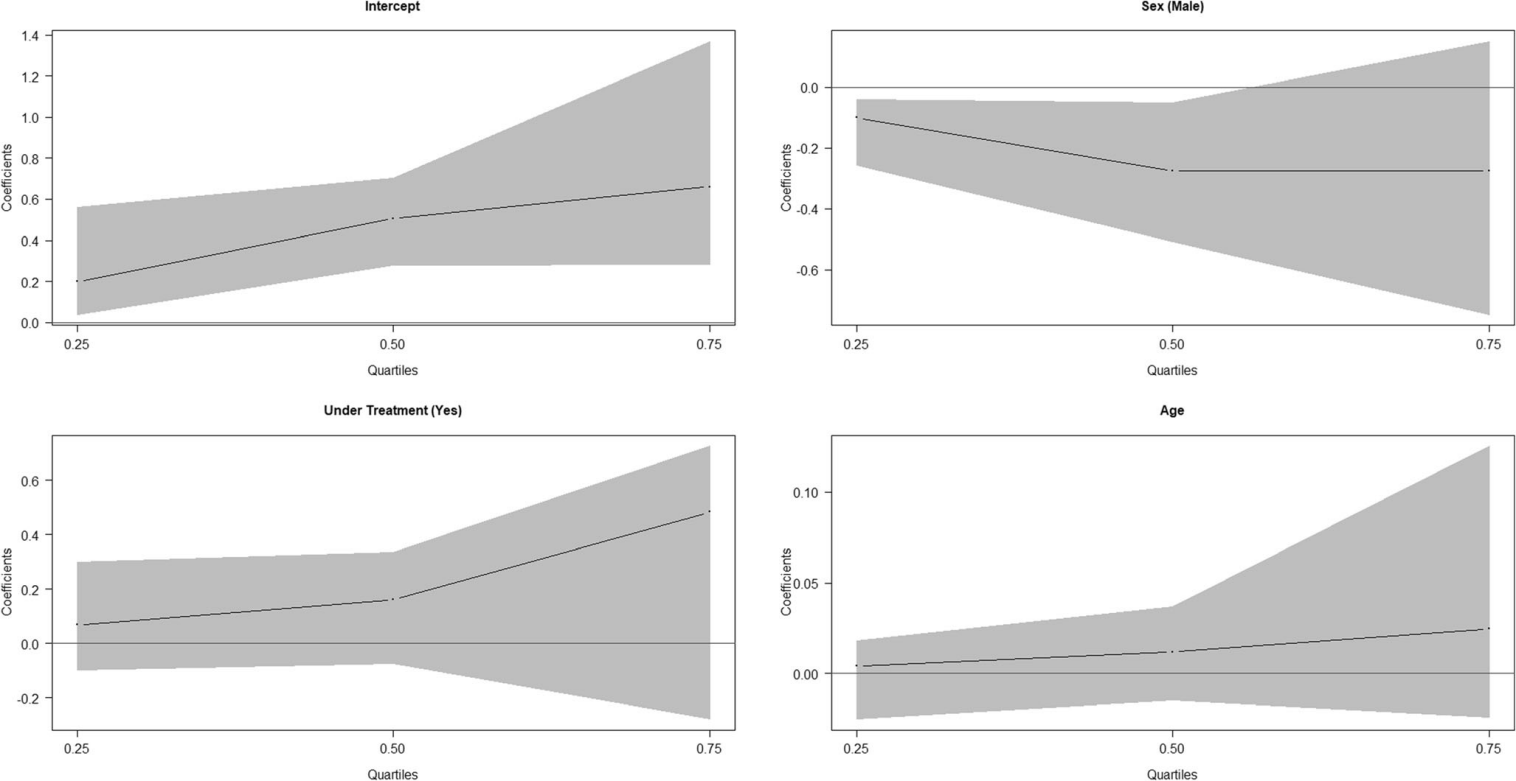
Quantile regression results for the absolute value of the difference between Demirjian’s estimated age and chronological age in subjects with RA

**Fig. 4:**
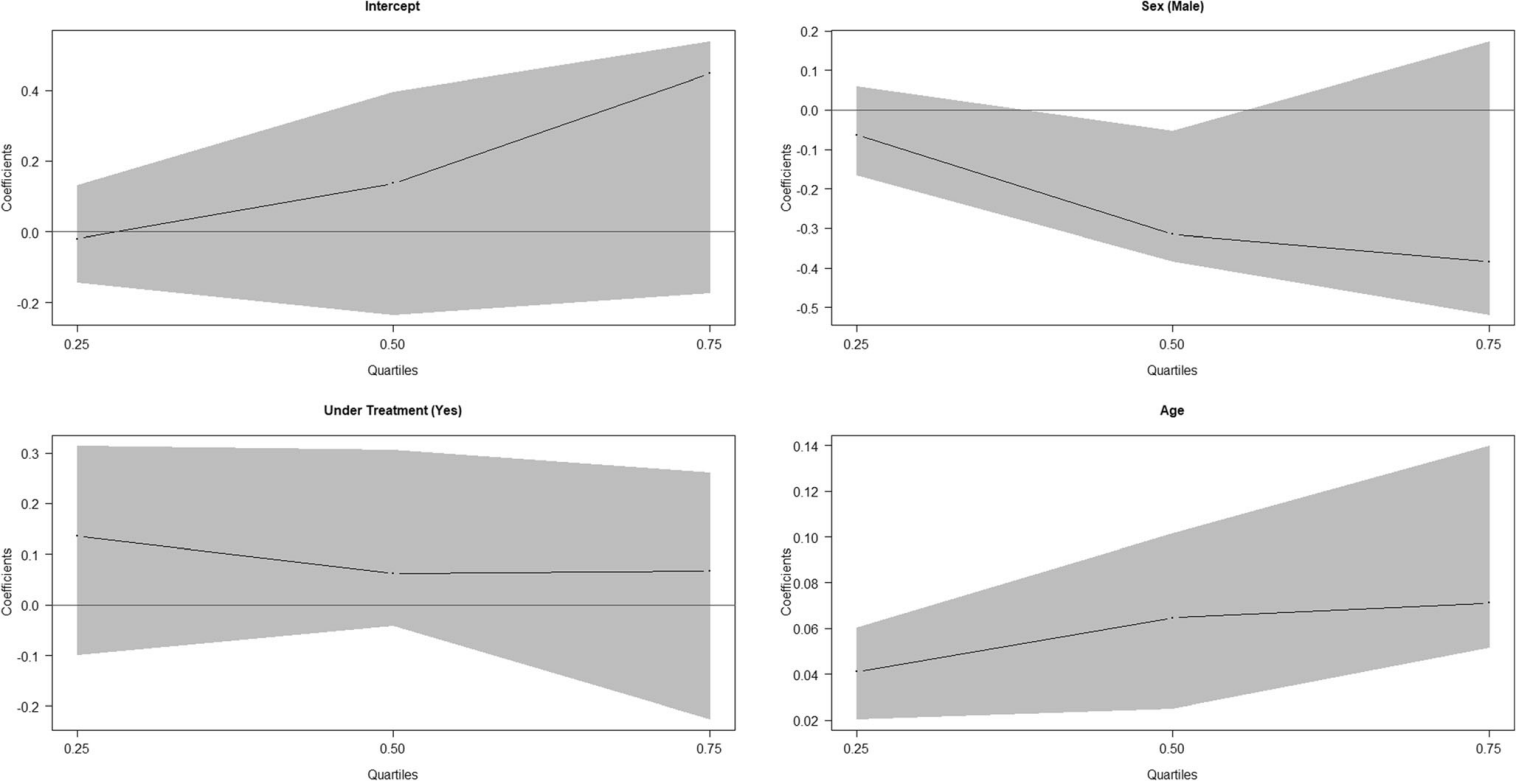
Quantile regression results for the absolute value of the difference between Willems’ estimated age and chronological age in JRA-affected children

The association of the absolute error with treatment in the JRA patients was analysed and is shown in Table 4 and Figs. 3 and 4. According to the results obtained applying the Demirjian’s method (Fig. 3), no association was found between the treated and non-treated subjects in all the quartiles. In boys, the absolute error was significantly lower with both methods in the median and the first quartile for Demirjian’s method. Applying Willems’ method, age was positively and significantly associated with absolute error, and treatment seemed not to affect the absolute error.

**Table 4:** Estimation results of the quantile regression for the absolute value of the difference between Demirjian and Willems’ estimated age and chronological age in subjects with JRA

		Demirjian	Willems
Quantile	Variable	Coef. (CI 95%)	Coef. (CI 95%)
tau = 0.25	Sex (male)	− 0.100* (− 0.257; − 0.040)	− 0.064 (− 0.164; 0.060)
	Under treatment (yes)	0.069 (− 0.097; 0.299)	0.136 (− 0.098; 0.313)
	Age	0.004 (− 0.025; 0.018)	0.041* (0.021; 0.060)
	Sex (male)	− 0.276* (− 0.508; − 0.050)	− 0.315* (− 0.383; − 0.054)
tau = 0.50	Under treatment (yes)	0.161 (− 0.073; 0.337)	0.062 (− 0.041; 0.306)
	Age	0.012 (− 0.015; 0.037)	0.065* (0.025; 0.101)
	Sex (male)	− 0.276 (− 0.747; 0.149)	− 0.385 (− 0.518; 0.173)
tau = 0.75	Under treatment (yes)	0.483 (− 0.279; 0.726)	0.068 (− 0.226; 0.261)
	Age	0.025 (− 0.024; 0.125)	0.071* (0.052; 0.140)

Table 5 shows the age estimation error obtained for a sample of healthy children compared with a JRA-affected children sample, both featured with an identical span of chronological age. The summary statistics of the errors in this balanced sample are reported by sex and presence of JRA. As it is shown in Panel A, Willem’s method underestimated chronological age in girls with a statistically significant difference compared with the Demirjian’s method, while no difference emerged between the methods for boys. The *p* values in a row showed that there were differences between girls and boys in terms of age estimation accuracy either with Demirjian or Willems’ method. Table 5 further showed that the methods estimated age differently both in healthy and in JRA-affected children (Panel A).

**Table 5:** Summary statistics of the difference and the absolute value of the difference between the estimated age and the chronological age in the balanced sample

Panel A: age estimation error, *E*					
Variable		*n* (%)	Demirjian mean (s.d.)	Willems mean (s.d.)	*p* Value
Sex	Female	265 (54)	0.05 (1.63)	− 0.31 (1.65)	< 0.01
	Male	228 (46)	0.56 (1.57)	0.52 (1.73)	0.47
		*p* Value	< 0.01	< 0.01	
Presence of rheumatoid arthritis	Yes	60 (12)	0.54 (1.07)	− 0.31 (1.22)	< 0.01
	No	433 (88)	0.25 (1.68)	0.13 (1.79)	< 0.01
		*p* Value	0.07	0.02	
Panel B: absolute age estimation error, |*E*|					
Variable			Demirjian median (1st–3rd quartile)	Willems median (1st–3rd quartile)	*p* Value
Sex	Female		0.97 (0.48–1.80)	1.13 (0.50–1.88)	0.10
	Male		1.05 (0.47–1.93)	1.03 (0.53–1.96)	0.03
		*p* Value	0.68	0.64	
Presence of rheumatoid arthritis	Yes		0.68 (0.26–1.05)	0.71 (0.48–1.39)	0.40
	No		1.08 (0.50–1.92)	1.14 (0.52–2.02)	< 0.01
		*p* Value	< 0.01	0.01	

The *p* value refers to the significance of a *t* statistics in panel A and to a significance of Wilcoxon statistics in Panel B

Panel B of Table 5 reports the absolute errors of chronological age compared by sex and presence of JRA. When comparing the results by sex, the methods were statistically different in terms of absolute error only for boys. On the contrary, the analysis of absolute errors showed a statistically significant difference among JRA-affected and non-affected subjects. Interestingly, both methods exhibited a lower median absolute error in the presence of JRA.

**Fig. 5:**
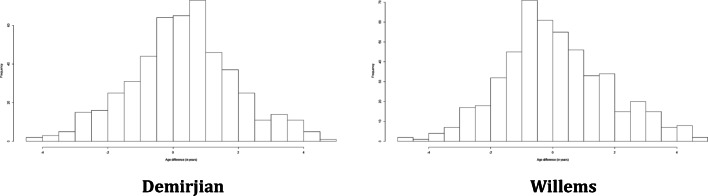
Distribution of the error in age estimation stratified by method of age estimation in the balanced sample

Figure 5 reports the distribution of the error in the balanced sample stratified by the method applied for age estimation. As in the case of the affected sample, the errors seemed to be normally distributed, which was again confirmed by the non-rejection of the null hypothesis of the Kolmogorov-Smirnov test, whose *p* values were 0.41 and 0.18 in case of Demirjian and Willems’ methods, respectively. For this reason, we applied a linear regression to analyse the association of the error with sex, the presence of JRA and chronological age in Table 6.

**Table 6:** Estimation results of the linear regression for the difference between estimated age and chronological age using Demirjian and Willems’ methods

	Demirjian		Willems	
Variable	Coef. (std. error)	*p* Value	Coef. (std. error)	*p* Value
Sex (male)	0.604* (0.143)	< 0.01	0.822* (0.155)	< 0.01
Rheumatoid arthritis (yes)	0.381 (0.218)	0.081	− 0.235 (0.236)	0.146
Age	− 0.186* (0.032)	< 0.01	− 0.086* (0.035)	0.014

The dependent variable in the linear regression in Table 6 was the estimation error in the balanced sample (*n* = 493), while the covariates were sex (with males as reference), presence of JRA (with presence of JRA as reference), and age. It is worth to be noticed that, in the case of Demirjian’s method, the age was significantly overestimated of approximately 0.6 years in boys. The presence of JRA did not significantly affect the estimation error with Demirjian’s method. Conversely, we found an age underestimation of about 0.186 years for any year increasing of the subject’s age. The analysis of the Willems’ method underlined that age was significantly overestimated in boys and underestimated as age increases. The presence of JRA did not affect the estimation.

**Fig. 6:**
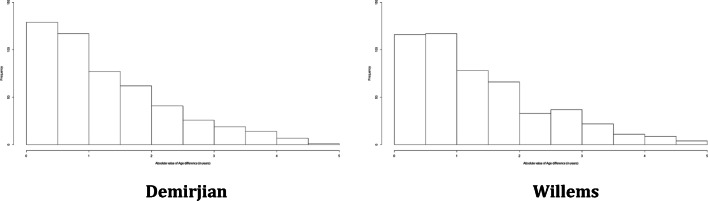
Distribution of the absolute value of the error in age estimation stratified by method of age estimation in the balanced sample

Since the distribution of the absolute error was highly asymmetric and not normal, as shown in Fig. 6, we assessed its association with the same covariates used in Table 6 through a quantile regression.

**Fig. 7:**
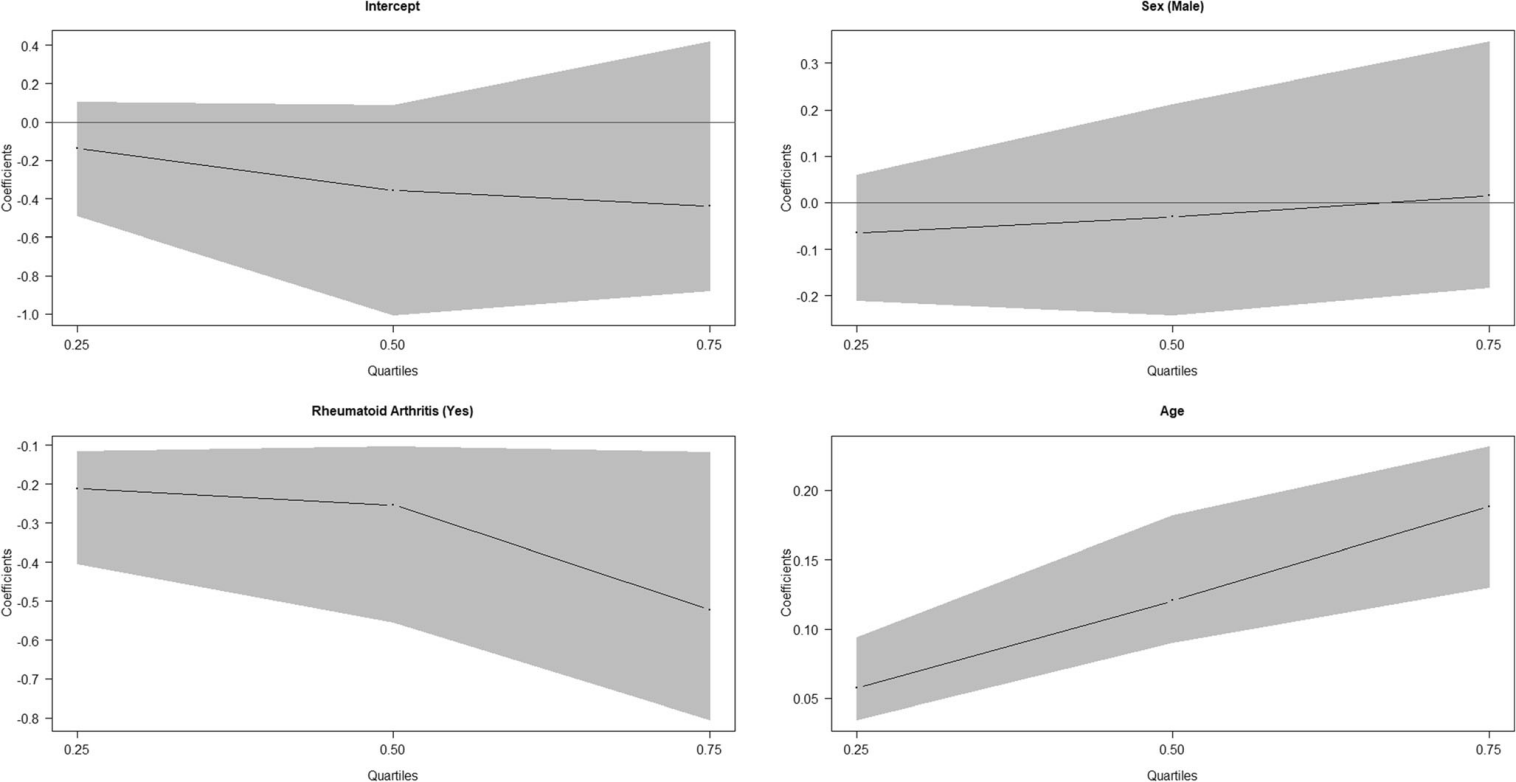
Quantile regression results for the absolute value of the difference between Demirjian’s estimated age and chronological age

**Fig. 8:**
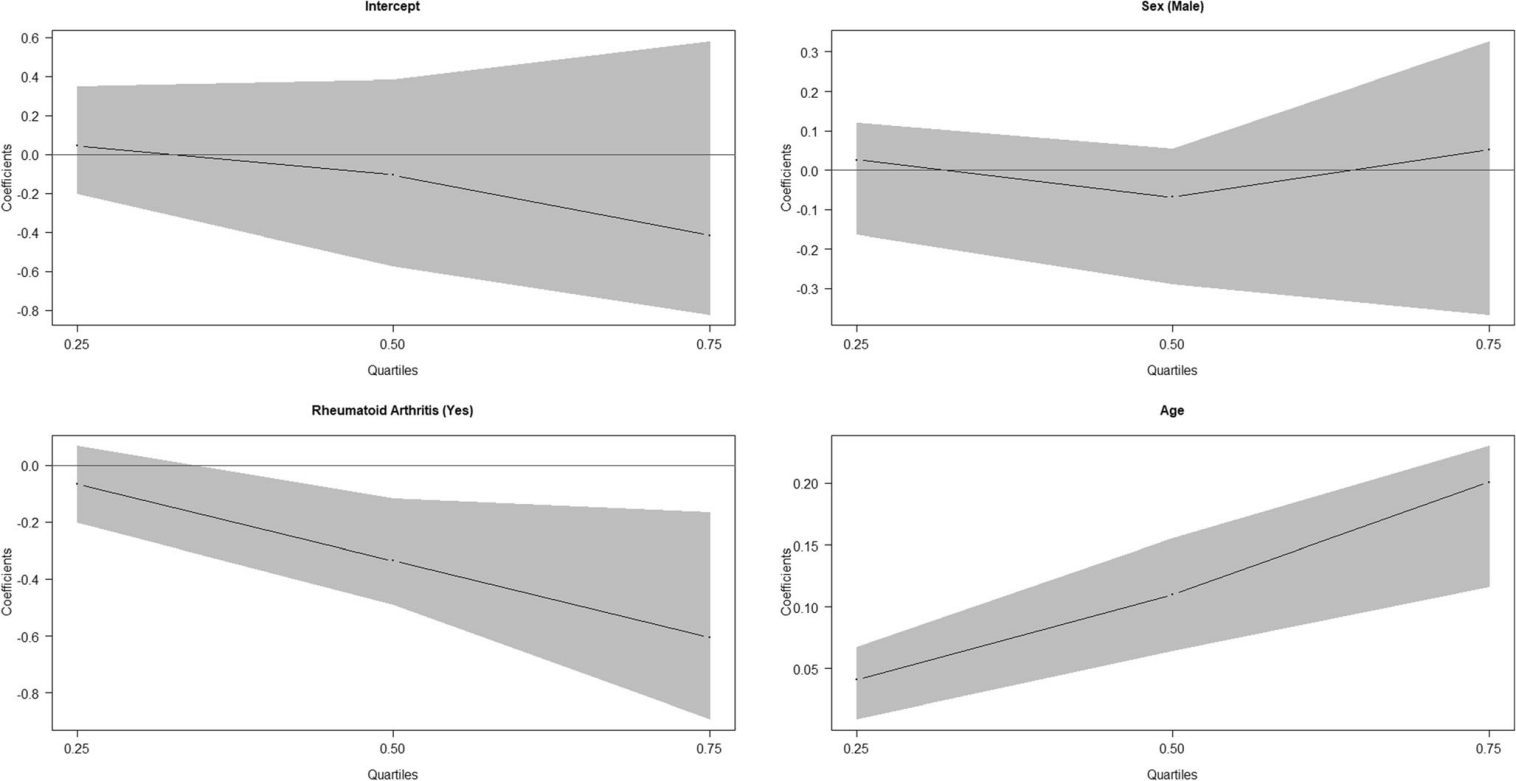
Quantile regression results for the absolute value of the difference between Willems’ estimated age and chronological age

From Table 7 and Fig. 7, it could be deducted that the absolute error made with Demirjian’s method was positively and significantly associated with the age of the subjects for each of the considered quartiles. This means that the error grows as age increases. Moreover, in the JRA-affected subjects, the absolute error was significantly lower than in healthy subjects and this was true for all the quartiles. The results obtained with the Demirjian’s method also underlined that the absolute error was not gender related. Table 7 and Fig. 8 show that the absolute error in estimation with Willems’ method was also positively associated with the chronological age of the subject. As in the case of the Demirjian’s method, the absolute error tended to be significantly lower in presence of JRA in the second and third quartile.

**Table 7:** Estimation results of the quantile regression for the absolute value of the difference between Demirjian and Willems’ estimated age and chronological age

		Demirjian	Willems
Quantile	Variable	Coef. (CI 95%)	Coef. (CI 95%)
tau = 0.25	Gender (male)	− 0.065 (− 0.211; 0.059)	0.026 (− 0.162; 0.120)
	Rheumatoid arthritis (yes)	− 0.212* (− 0.404; − 0.115)	− 0.067 (− 0.202; 0.066)
	Age	0.057* (0.034; 0.094)	0.041* (0.009; 0.067)
tau = 0.50	Gender (male)	− 0.031 (− 0.241; 0.212)	− 0.069 (− 0.288; 0.054)
	Rheumatoid arthritis (yes)	− 0.254* (− 0.553; − 0.104)	− 0.336* (− 0.487; − 0.117)
	Age	0.121* (0.090; 0.182)	0.110* (0.065; 0.155)
tau = 0.75	Gender (male)	0.015 (− 0.182; 0.347)	0.051 (− 0.366; 0.326)
	Rheumatoid arthritis (yes)	− 0.523* (− 0.805; − 0.118)	− 0.604* (− 0.891; − 0.167)
	Age	0.188* (0.130; 0.231)	0.201* (0.117; 0.230)

*states a statistically significant effect

## Discussion

The age estimation procedure is a difficult task involving legal, judicial and social issues of such an utmost importance that only highly reliable methods are required to be applied. In the assessment procedures which lead to the age estimation result, all the useful biological and documentary evidences must be probed and taken into account.

The analysis of the scientific literature allows us to say that in dental age estimation procedures, the Demirjian 8 stages classification of dental development is one of the most applied and adopted methods [12, 13].

The skeletal system and the teeth—biological structures of different embryologic derivation—are the body districts more useful for auxological and forensic age evaluation in children. Vast literature exists which describes how and how much the skeletal growth is affected, though by nutritional and even socioeconomic conditions, HIV positiveness, congenital chromosomic and metabolic anomalies and systemic illnesses.

Very few studies exist, however, which test the influence of systemic disorders on the dental mineralization process. Generally speaking, the dental development (mineralization and crown-root formation) process is considered unaffected by even severe illnesses, such as syndromic pathologies [14].

The process of dental mineralization is therefore considered as very stable and uninfluenced by pathologies (congenital, hormonal, etc.) or alterations of overall growth, but specific literature deals with the pathologic conditions that can affect tooth calcification. Although specific studies for the systemic pathologies are necessarily limited to a few subjects, given the high difficulty in collecting the study cases, some congenital syndromes [14], such as the epidermolysis bullosa [15] and the X-trisomic syndrome [14] seem not to affect tooth mineralization. On the contrary, in pathologies such as amelogenesis imperfecta [16], Turner and X-fragile syndromes [17], an advanced root mineralization has been noticed, while hypodontia [18, 19], Apert syndrome [20] and thalassemia major [21] resulted in a simple slowing effect on it. It is also reported that severe hormonal diseases may affect the tooth development process, but data in the literature are controversial about the point (in growth-hormone deficiency-affected individuals [22–24], which seems to happen at a slower dental development but data about the entity of such an illness and of the possible normalization effect of a hormonal substitutive therapy are still not homogeneous).

Our present study focuses on juvenile rheumatoid arthritis (JRA), a major paediatric rheumatic disease of still unclear origin, characterised by chronic inflammation in multiple tissues, including the joints, tendon, muscles and several visceral organs [25, 26]. Non-steroidal anti-inflammatory drugs (NSAIDs) are usually chosen as first-line therapy in the medical treatment of the disease. Immunosuppressive and immunomodulating agents are often added as a second-line medication to patients who unsatisfactorily respond to the NSAIDs therapy. The systemic corticosteroids have a role in initial treatment or bridging therapy when sufficient control over the inflammatory process cannot be adequately achieved with NSAIDs or immunosuppressive drugs [6, 25]. However, a prolonged use of systemic corticosteroids is sometimes inevitable in patients having a poor response or unpleasant side effects with NSAIDs or immunosuppressive drugs.

JRA invariably causes a delay in linear growth of the body as a well-recognised complication in children suffering from the disease [1, 2] so that the growth-inhibiting effects of both continuous corticosteroid therapy (protracted for more than 1 year) and underlying chronic illness such as JRA are well recognised as well [3–8]. According to our knowledge, only very few previous researches investigated the influence of JRA on dental maturation.

Ley et al [27] examined 64 OPGs of children affected by JRA and aged between 6.7 and 14.4 years. The mean values of each age groups resulted to fall in the maturation curves reported by previous studies for healthy Canadian, German and Dutch children. The authors concluded that the examined affected sample showed neither an advanced dental maturity nor any alteration of the dental maturation process.

Lehtinen et al [28] applied Demirjian’s 8 stages method on 168 affected Finnish subjects with chronological age from 6.3 to 14.4 years compared with an equal sample of healthy children. Most part of the discussion focused on the influence of steroid therapy on dental maturation since the authors found an acceleration of root mineralization while the long bone development, mediated by endochondral cartilage, was delayed by corticosteroid administration in JRA-affected children. The mean difference between dental age and chronological age was lower in healthy subjects as compared with patients affected by JRA and this accelerated root maturation was attributed to the steroid therapy and not to the JRA per se.

The first relevant finding that can be drawn from the present research—even if based on a small sample—is that Willems’ and Demirjian’s methods proved to be accurate in providing dental age estimations both in healthy children and in children affected by JRA. No significant differences of estimates emerged between sexes and the treated and non-treated subjects affected by JRA; neither sex, steroid therapy nor the chronological age resulted to be significantly associated with error in a linear regression framework. In terms of accuracy, Willems’ method tended to underestimate age while Demirjian’s method tended to overestimate it, both for treated and non-treated JRA subjects. The fact that the two methods are based on the same classification of dental maturation (original 8 stages Demirjian’s grading system) allows to infer that the statistically significant difference detected between the two sets of estimates are likely to be due to the different conversion tables and the interindividual variability rather than to an influence of JRA. The quantile analysis of absolute errors of the JRA-affected sample revealed that the Demirjian’s method produced more precise estimates in boys than girls, while the precision of Willems’ method decreased as the chronological age increased. In any case, the analysis revealed no differences between treated and non-treated subjects; thus, a systematic effect of steroid therapy on dental maturation process can be excluded. In disagreement with Lehtinen’s report, the latter findings do no support the conclusion that corticosteroid treatment influences the pattern of root calcification.

The comparison of JRA sample with healthy children firstly confirmed that the Demirjian or Willems’ methods estimated age differently in the healthy and JRA-affected children. Demirjian’s method confirmed to overestimate chronological age while Willems’ method tended to underestimate and produce an increasing error size as the chronological age of the subject increases. The analysis of absolute errors showed a statistically significant difference among JRA-affected and healthy subjects, being the median errors significantly lower in the affected compared with the healthy children. This result is unexpected, but only apparently confounding. From a biological point of view, it is not credible that in JRA-affected children, dental maturation occurred more evenly and more aligned with the predictions obtained applying methods based and tuned for healthy children (Demirjian and Willems in this case). On the contrary and given the limited size of both samples considered here, it is possible that the interindividual variability resulted by chance lower in the affected compared with the healthy sample.

Moreover, the findings of the research presented here do not confirm Lehtinen et al.’s [28] conclusions about the possible acceleration of root mineralization due to corticosteroid therapy compared with non-treated JRA-affected children and to healthy subjects.

On the other hand, the present results are in agreement with those reported by Ley et al [27] who found no alterations of the dental maturation pattern in JRA-affected children. Nevertheless, it is to be noticed that these authors offered no direct comparison with a sample of healthy individuals but referred to previous large studies that reported the age estimation performances described by the original study of Demirjian or more recent researches on Dutch or German children. Given the limited sample, the authors could not conclude about the influence of steroids on dental maturation, suggesting also to consider methotrexate, whose effect on dental maturation was not studied.

## Conclusions

The JRA and its steroid therapies can deeply affect the skeletal developmental pattern, one of the most important biological evidences, making it unreliable or less reliable for chronological age estimation. The present findings show that dental development is a stable process which is not influenced by JRA or steroid therapies. A previous study reported an advanced dental root maturation in JRA-affected children treated with steroids, but this trend is not confirmed here. Our research is a contribution to the study of the factors possibly affecting dental development (diseases, nutrition, geographical residence, ethnicity, etc.) and of the reliability of the currently applied methods of age estimation. The less a biological evidence is actually influenced by any factors, the larger is its reliability and should be the expert confidence in its application; moreover, the application of such a reference parameter (dental maturation) should be more recommended as far as the reliability of the only other applicable biological evidence (i.e. skeletal development) is affected by diseases or other external factors. Beyond this general scientific value, the results of the present research show that the methods and conversion tables developed and commonly applied for age estimation in healthy subjects can be usefully applied also in JRA-affected individuals. It is worth noting, moreover, that the JRA-affected subjects, contrarily to other individuals affected by chromosomal diseases, have no psychic or psychological impairments which would actually render useless the judiciary age estimation assessment.

## Electronic supplementary material

ESM 1 (DOCX 24.1 KB)
