# Supplementary material for: Dental age estimation in children affected by juvenile rheumatoid arthritis
Source: Int J Legal Med. 2020 Aug 20;135(2):619–29. doi: 10.1007/s00414-020-02395-w (PMC7870602; doi:10.1007/s00414-020-02395-w)
Supplement: Supplementary file 1 — (DOCX 24.1 KB) [file 414_2020_2395_MOESM1_ESM.docx]

| **Table A1.** Distribution of age stratified by presence of JRA and treatment – Original sample (without repeated measures) | | | |  |
| --- | --- | --- | --- | --- |
| **Age (in years)** | **Without JRA** | **With JRA** | | |
|  |  | Treated | Not treated | |
| **3** | - | - | 4 | |
| **4** | - | 3 | 10 | |
| **5** | - | 2 | 6 | |
| **6** | 5 | 4 | 4 | |
| **7** | 12 | 2 | 3 | |
| **8** | 18 | 7 | 6 | |
| **9** | 50 | 1 | 4 | |
| **10** | 74 | 6 | 6 | |
| **11** | 100 | 3 | 7 | |
| **12** | 98 | 5 | 2 | |
| **13** | 85 | 4 | 5 | |
| **14** | 106 | 1 | 4 | |
| **15** | 101 | 2 | 2 | |
| **Total** | 649 | 40 | 63 | |

| **Table A2.** Distribution of age stratified by presence of JRA and treatment – Balanced sample | | | |  |
| --- | --- | --- | --- | --- |
| **Age (in years)** | **Without JRA** | **With JRA** | | |
|  |  | Treated | Not treated | |
| **6** | 5 | 1 | 1 | |
| **7** | 12 | 2 | 1 | |
| **8** | 18 | 1 | 2 | |
| **9** | 33 | 1 | 4 | |
| **10** | 74 | 6 | 6 | |
| **11** | 66 | 3 | 7 | |
| **12** | 60 | 5 | 2 | |
| **13** | 62 | 4 | 5 | |
| **14** | 58 | 1 | 4 | |
| **15** | 45 | 2 | 2 | |
| **Total** | 433 | 26 | 34 | |
